# A search tool based on language modelling developed for The Index of Middle English Prose

**DOI:** 10.12688/openreseurope.16590.2

**Published:** 2024-03-11

**Authors:** Alpo HONKAPOHJA, Jacob Thaisen, Anders Nøklestad

**Affiliations:** 1 Department of Literature, Area Studies and European Languages, University of Oslo, Oslo, Oslo, 0315, Norway; 2 Humit, University of Oslo, Oslo, Oslo, 0313, Norway

**Keywords:** Language modelling, Middle English, medieval studies, bibliography, ngrams, digital humanities, historical linguistics

## Abstract

Non-standardised early vernaculars present a problem for search tools due to the high degree of variation. The challenge lies in the variation found in orthography, syntax, and lexicon between titles, incipits, and explicits in manuscript copies of the same work. Traditional search methods relying on exact string matching or regular expressions fail to address these variations comprehensively. This project presents a web-based search tool specifically designed to handle linguistic and textual variation. The software is made available as a part of the *Index of Middle English Prose* (IMEP).

The search tool addresses the issue of variation by utilizing a database of incipits and explicits, character-based n-gram language models (LMs) built with the *Stanford Research Institute Language Modelling* (SRILM) toolkit, and a fuzzy search script (IMEP: FSS) written in Python. The tool optimizes for recall, retrieving multiple potential matches for a search string, without attempting to identify the ‘correct’ one. The search process involves looking up exact matches in the database while simultaneously using the fuzzy search script to evaluate the incipits and explicits against a model of the search string, followed by a match of the search string against models of the incipits and explicits. This two-step process shortens the processing time, which would otherwise be unreasonably long, because while using SRILM to match the search string against each incipit or explicit in the IMEP for precision could be time-consuming, running a first step where all texts are matched against a single LM built from the search string allows for faster processing.

A web application, built using Django and Docker, combines the results of the direct database lookup and the fuzzy search script, presenting them as a list with exact matches followed by fuzzy matches ordered by increasing model perplexity. The tool is made available Open Access and can be adapted to other datasets.

## Introduction

Access to medieval manuscripts, which form a major part of our collective heritage, is dependent on the search tools available to us. The *Index of Middle English Prose* (IMEP) seeks, for the first time, to locate and identify all surviving English prose texts composed between ca. 1200 and 1500, through its printed catalogues and website. IMEP entries record the opening lines (incipits) and closing lines (explicits) of texts that have been indexed so far. A crucial weakness of the IMEP is the lack of a satisfactory digital search tool for interested parties to search the collection, especially since the database records preserve linguistic variation inherent to a non-standardised vernacular like Middle English as well as textual variation inherent to manuscript copies of one and the same work. The challenge lies in the fact that records pertaining to a single work will differ in orthography, syntax, and lexicon, and this will be true of the title as well as the incipits and explicits. To address this challenge, the present paper focuses on developing a language modelling-based tool capable of handling the diverse forms of variation.

The IMEP is the main reference work for non-verse texts written in Middle English (hence ME). The groundwork for this project was laid over 40 years ago, in 1978, at a conference in Cambridge called *Problems in Middle English Prose* ( *cf.* Edwards & Pearsall, 1981). The first three volumes were published in the mid-1980s ( Hanna, 1984; Horner, 1986; Lester, 1985), and as of today, there are 24 published print volumes with several more in progress. This project stands as the most important resource for uncovering crucial insights into patterns of textual transmission in medieval England. The availability of a comprehensive index will greatly benefit scholarship, providing a complete overview of existing texts and works. Otherwise, there is a risk that scholarly attention will be disproportionately focused on edited works. As Jolliffe (1974: 12) observed: “[s]ometimes the reader is left with the impression that apart from these works, no ME […] writings existed”.

**Figure 1.**
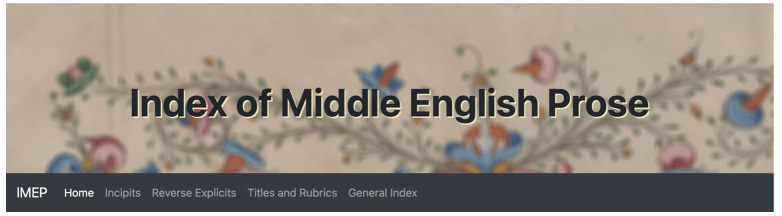
IMEP user interface.

The publication of IMEP volumes has proceeded collection by collection with the publication of handlists, which usually cover a single collection in a single repository (sometimes several repositories are covered by a single volume, such as volume 15 on Midland libraries; at other times, with large libraries such as the British Library or the Bodleian, only one collection in the repository is covered). However, an online version of the IMEP is maintained at Cambridge University Library (CUL), which makes searchable, respectively, Incipits, Reverse explicits, Titles and rubrics and a General index (see Figure 1).

The basis for making queries in the IMEP search tool is a database of **incipits** and **explicits**. The database was compiled as a part of the IMEP handlists.

**Incipit** (Latin: 'here begins') can be defined as "[T]he opening word or words of a medieval Western manuscript or early printed book" ( Encyclopædia Britannica).

**Explicit** (Latin 'here ends'): refers to the closing words of a medieval manuscript of early printed book, frequently concluding with the words *explicit* or *amen*.

The website offers, at the moment, a simple text-based search and a regular-expressions based one. Our tool is designed to supplement these options through offering an enhanced search function for the incipits and explicits, which are recorded with all their linguistic variation intact.

### Variation in Middle English

The purpose of the IMEP is to aid researchers in identifying a text as being a copy of a particular work, and to aid them in identifying other extant texts of the same work. However, one of the challenges that arise from this process is the amount of linguistic variation found in ME. This period is known for its lack of a standardized writing system, resulting in a wide range of variations in the written language. Consequently, such variation poses a difficulty for search tools. The IMEP does not normalise the language of the incipits and explicits of the items it records but preserves them with much of the original variation intact, which is so characteristic of ME and other early vernaculars. While the indexers have varied in the number of words they have recorded, they have adhered to the same transcription guidelines, which increases comparability.

ME, which is defined in the IMEP as English texts written between 1200 and 1500, is characterised by linguistic variation. In the words of Benskin *et al*. (2013: §1.1.2):

*Middle English represents that stage in the history of the language most highly characterised by diversity of written form; while dialects have been spoken at all periods, it is only during late mediaeval times that local usage is regularly reflected in writing.*

The challenge of working with ME lies in the fact that variation exists not only in spelling but also in syntax and lexicon and in the title as well as incipits and explicits. Table 1 shows a small sample of different incipits of the ME prose *Brut* chronicle, which is one of the most widely surviving ME works. Matheson (1998) lists 181 manuscripts.

**Table 1.** Examples of incipits of the prose *Brut* chronicle: [In the noble land of Syria, there was a noble king].

Incipit	IMEP volume
In the nobele lande of syrrye there was a nobele kyng and	Rylands Eng 103 [1]
In the noble land of surey þer was a noble king	BL Add 10099 [1]
In the noble land of surre ther was a noble kyng and myhty and a	Lambeth 84 [1]
Off the noble land of surrye ther was a royal kynge	Peniarth 343 [1]
In the noble land of surrye ther was a worthi kynge	Lei UL 47 [1]
Some tyme in the lande of surre ther was a myghty & a ryall	Oxf Un 154 [1]
In the noble londe of surrye was some tyme a grete kynge	Lambeth 306 [4]

As Table 1 shows, variation can occur on many different levels, including spelling ( *there* vs. *þer*, *syrrye* vs. *surey* vs. *surre* vs. *surrye*), lexicon ( *nobele kyng*, *royal kynge*, *worthi kynge; In the noble land vs. off the noble land*), and word order; and noun phrases can be incomplete ( *a noble kyng and myhty* vs. *a myghty & a ryall* [king]). What is more, the number of words recorded in an incipit may vary, because IMEP is based on handlists collected and edited by different scholars, who may have recorded slightly different length of text.

It is all this variation that our search tool needs to be able to handle. However, before presenting our tool we need to discuss other available approaches for searching ME texts.

### Alternative approaches

***Simple search*.** Various solutions are available for locating words and phrases within ME texts. The most basic approach involves a simple search for an exact string, which is used by the IMEP website (in addition to offering a regular expressions-based search). The advantage of this approach is its straightforward nature. Consequently, the simple searches are best suited for conducting searches in modern standardized language. For instance, the Titles and the General index on the IMEP website, which are recorded in their modern forms, allow users to search for specific works like copies of the ME prose *Brut* by simply entering "Brut" in the Titles index. However, for the other indexes that are not in modern forms, this method relies on users’ familiarity with common ME spelling variants, requiring them to manually input those variants. As a result, users will typically retrieve only a subset of the relevant matches from the database they are searching. For example, searching for the string "Syria" SYRIA would fail to find spellings such as *syrrye*, *surey*, *surre* or *surrye* (as listed in Table 1).

Another option is to have a highly annotated or well-structured dataset. For instance, *the Digital Index of Middle English Verse* (DIMEV) enables users to search for shelf-marks, verse forms, and other meta-information, which have all been recorded in its database. However, users cannot search for titles, incipits, or explicits in normalized or non-normalized form; they can only browse through an alphabetically ordered list of them. It is important to note that work for the *Index of Middle English Verse* ( *IMEV*) started already in the 1930s and has been completed ( *cf.* Brown & Robbins, 1943: v), while the IMEP is still a work in progress and the plan is only to add features such as numbering for ME works and their different versions once all the handlists have been completed. Consequently, the identification process for all extant works in IMEV is completed, whereas for the IMEP it is still work in progress.

***Regular expressions*.** A common approach to enhancing search capabilities is the use of regular expressions. Examples of this can be found in the IMEP website and the *electronic Voigts and Kurtz* (eVK2). However, regular expressions often fall short of achieving the desired results, as they assume users can predict every conceivable linguistic variation.

A typical regular expressions approach would be to search for the string “k[iy]ng” to retrieve instances of both *king* and *kyng*, but the extent of spelling variation far surpasses these common forms. For example, the *Middle English Dictionary* (MED) records not only the Present-Day English (PDE) spelling *king*, but also the alternative forms *kingue*, *kingk*, *kink*, *ging*, *keng*, *keing*, *kining*, *cining*, *cing*, *cinȝ*, *chinge*, *chinȝ* and more (but neither *kyng* or *kynge*, *cf.* Table 1 above). The difficulty of accurately predicting spellings multiplies when expanding the search to other lexical items. Searching for variants like *nobel*, *noble*, *nobill*, *nobele*, *nooble* and *royal*, *ryall* would require complex rules to interchange *oya* with *ya*, *o* with *oo*, *el* with *le* and *ele*, and *be* with *bi*, as well as accounting for variations like *l* versus *ll*. All this would add up in complex query strings that users would struggle to produce or input accurately.

Another difficulty with regular expressions is that variation in word order is difficult to predict. Consider these examples from Table 1:

***Some tyme** in the lande of surre ther was a myghty & a ryall* (Oxf Un 154 [1])

*In the noble londe of surrye was **some tyme** a grete kynge*. (Lambeth 306 [4])

The position of the prepositional phrase ‘some time’ differs in these instances, beginning the first one and following the verb *was* (TO BE) in the second one. Moreover, unexpected lexical variants such as ‘worthy king’, ‘great king’ or ‘royal king’ would not be accurately predicted when searching for variant spellings of ‘noble king’.

A variant of the regular expressions approach is to take the task of generating query strings out of the individual user’s hands. Software can apply regular expressions to extensive sets of attested spelling forms for lemmata, such as those recorded by the *Oxford English Dictionary* (OED), *MED*, *Linguistic Atlas of Early Middle English* (LAEME), and other reference works to generate even larger sets of conceivable forms. Texts can then be searched for these generated forms. However, this approach will generate a large number of spurious forms for each lemma and does not guarantee an exhaustive identification of all relevant forms. Moreover, it is better suited for single-word lookups in a dictionary, whereas IMEP is designed to be used for the identification of opening or closing lines consisting of several words.

***Normalization*.** Another approach involves the normalization of spellings, a process that entails transforming variant spellings into a standardized form. One well-known tool that relies on this is approach is VARD2, which is designed to normalize spelling variations in Early Modern English (EModE, ca. 1500–1700). VARD2 has gained considerable traction within the field of Early Modern English corpus linguistics and has been utilized in various projects. Examples of such projects include *Early Modern Medical Texts* (EMEMT), *Innsbruck Computer Archive of Machine-Readable English Texts* (ICAMET), *The Lampeter Corpus of Early Modern English Tracts* ( *cf.* Schmied, 1994), *A Corpus of English Dialogues 1560–1760* and *ARCHER: A Representative Corpus of Historical English Registers*.

VARD2 operates by matching EModE spellings with “a large modern dictionary or word list derived from the *British National Corpus* (BNC)” ( Archer *et al.*, 2015: 9). It accomplishes this by using a list of formerly known variants, a set of letter replacement rules, phonetic matching by the SoundEx algorithm and the Levenshtein edit distance metric ( *cf.* Archer *et al.*, 2015: 9–10; Rayson *et al.*, 2007: 2). However, it is important to note that the tool is tailored for EModE, a period with a relatively more confined degree of linguistic and lexical variation compared to ME. Furthermore, there is less lexical overlap between Medieval and Modern English, which means that approaches involving the matching of words with a word list from the BNC are less likely to be successful.

***Similarity and dissimilarity matrixes*.** There are also other digital solutions that struggle to address the extensive linguistic variation found in ME. In particular, a family of solutions apply standard distance metrics to compare two strings, such as edit distance (similarity matrix) or Jaccard distance (dissimilarity matrix). Edit distance metrics, used by tools like VARD2 (Levenshtein distance is a specific instance of edit distance), indicate how similar two strings are to one another. A search returns matches that are within a set distance away from a query string. However, applying this method to ME causes difficulties. These are illustrated in Table 2 and discussed below.

**Table 2.** Edit distance.

**A**	i	n		t	h	e		n	o	b	e	l	e		l	a	n	d		e		o	f		s	y	r	r	_	y	e		t	h	e	r	e		w	a	s		a		n	o	b	e	l	e		k	y	n	g		a	n	d
**B**	i	n		t	h	e		n	o	b	_	l	e		l	a	n	d		_		o	f		s	u	r	_	e	y	_		_	þ	e	r	_		w	a	s		a		n	o	b	_	l	e		k	y	n	g		_	_	_

The first problem encountered is how automatically to delimit the scope of comparison – here, excluding the final word *and* in string A from the comparison with string B. While this may seem intuitively straightforward for a human, it poses computational challenges. Likewise, if string A consisted only of the string *nobele lande of surrye there was* due to textual damage, such as a tear in the manuscript, the edit distance would be unreasonably large unless non-corresponding parts of string B were automatically excluded.

A second problem stems from how edit distance metrics inadequately distinguish between spelling variations and lexical variations: the edit distance between *king* and *chinȝ* will be comparable to that between *nobele* and *royal* with two exact matches ( *in* vs *in* and *o__l* vs *o__l)*. However, only the second pair is lexically different. Consequently, this might lead to false positive results in searches. The problem is even great with lexical variations like NOBLE vs WORTHY vs ROYAL.

Furthermore, edit distance also becomes unreasonably large in cases of syntactic variation, like the instances where the phrase ‘some time’ appears at the beginning of one copy and after the main verb in another (see above).

One would have to normalize spelling forms prior to measuring edit distance if one wished to measure lexical edit distance only. And normalizing spellings goes against the editorial principles of the IMEP.

These examples demonstrate the limitations of some commonly used computational approaches when handling variation in ME. Overcoming these challenges necessitates more sophisticated search methods that incorporate algorithms and techniques tailored to linguistic variations found in ME.

## Methods and materials

The search tool developed for IMEP offers a solution to tackle the challenges posed by the extensive linguistic and textual variation within ME texts. The tool addresses these challenges by utilizing a database of incipits and explicits, character-based n-gram language models (hence LMs) built with the *Stanford Research Institute Language Modelling* (SRILM) toolkit, and fuzzy search script ( IMEP: FSS) written in Python.

### The database of incipits and explicits

In IMEP, the explicits are recorded in a unique format known as reverse explicits. This means the words are recorded in reverse order (e.g. ‘amen scribe the of health the for pray us let story our ends here’). This convention is inherited from the printed handlists and enables human indexers to identify them as copies of specific works.

The database stores IMEP records in plain text, but encoded in a format that is TEI XML compliant. Word boundaries are indicated by <w/> milestone tags. ^1^


<w/> o w e n <w/> w e <w/> s o <w/> u s <w/> f o r &yogh; e u e <w/> g o d <w/> &thorn; a t <w/> w o l d e <w/> w e <w/> a s <w/> f o r <w/> m e r s i <w/> a n d <w/>


The average length of an incipit is 51 characters, while the explicits have an average length of 56 characters. Both figures include spaces between words. The data are, in other words, extremely sparse, providing limited information on which to base a measure of similarity between strings.

### The n-gram models

For making queries in the database, the search tool utilizes a set of character-based n-gram LMs. These models are created using the SRILM language modelling toolkit. SRILM is a toolkit primarily used for building and applying statistical LMs and has been used in various applications ranging from speech recognition to statistical tagging and segmentation, to machine translation (see Stolcke *et al*., 2011).

To execute the search processes, a fuzzy search Python script is utilized. The script assesses each LM against the query string and returns a perplexity of the model on the query string. For instance, the phrase, *in the nobele lande of syrrye there was a nobele kyng and* can be broken down into the following n-grams:

5-grams: #in#t, in#th, n#the, #the#, the#n, he#no, *etc.*

4-grams: #in#, in#t, n#th, #the, the#, he#n, e#no, *etc.*

3-grams: #in, in#, n#t, #th, the, he#, e#n, #no, *etc.*

2-grams: #i, in, n#, #t, th, he, e#, #n, no, ob, *etc.*

1-grams: #, i, n, t, h, e, o, b, l, a, d, *etc.*

(# represents a word boundary)

A 5-gram model of the phrase comprises a list of the 5-grams it contains along with their absolute frequencies and potential weights we introduce. A 4-gram model is a list of the 4-grams, accompanied by their absolute frequencies and possible weights, and this pattern continues for the shorter n-gram lengths. If another string (referred to as the **test text**, henceforth) is drawn from the same population (referred to as a **language**) as the modelled phrase (hence the **training text**), it will contain the same n-grams with the same frequencies.

Evaluation of these models is carried out through a metric called **perplexity**, which can be defined as the weighted average branching factor of a language (per n-gram). Perplexity, a commonly used intrinsic evaluation metric for n-gram LMs, numerically expresses the level of similarity between a test text and a model of a language built from a training text. This provides a basis for assessing whether they are samples of the same language. The lower the perplexity of a model on a query string, the more similar it is to that query string. Or, more accurately put, the more likely it is that the query string manifests the same language that the model is a model of.

The larger n-grams capture more of the lexicon, while the smallest ones capture individual characters. By using all five n-gram lengths, the tool takes into account both the spelling and the lexicon of the phrase. Moreover, the longest n-grams capture elements of word order by spanning the space between words. Importantly, they remain sufficiently short to prevent variations in word order from significantly raising model perplexity, which would be an undesirable effect.

### Calculating perplexity

The following discussion outlines the mathematical foundation behind the perplexity estimations. They are staples in language modelling, and do not originate with this project—we refer to Jurafsky and Martin (2008: 83–122) for a comprehensive introduction to the terms and steps outlined in the next paragraphs. They are provided here to illustrate and justify the specific settings. The underlying formula is a probability calculation and takes the following form.

$$P(\text{\#kyng\#}|\text{training}\;\text{text})=\frac{c(\#\text{ky})}{c(\#\text{k})}\times \frac{c(\text{kyn})}{c(\text{ky})}\times \frac{c(\text{yng})}{c(\text{yn})}\times \frac{c(\text{ng\#})}{c(\text{ng})}$$

Here, *P* represents probability, and *c* signifies the absolute frequency of the n-gram in the training text. The steps sketched out below involve not only summation over all relevant n-grams and interpolation of all n-gram lengths, but also manipulation of the numerator and the denominator to estimate a language from the sample that is the training text.

Consider the scenario where the character sequence (2-gram) is *#k*. In this context, the question is the probability of the next character (1-gram) being *y*. The fraction *c*(#ky)/ *c*(#k) expresses this probability. If the probability was 1:7, the corresponding perplexity would be 7. The complete formula, then, gives us the conditional probability, calculated at the 3-gram level only, for encountering the string *#kyng#* (the test text), the condition being the training text. This is achieved by multiplying together the probabilities returned by the individual fractions. Ultimately, it is the inverse of the average of those probabilities that is the perplexity for the string *#kyng#*. ^2^ As a first step in estimating the language from the training text, if the training text happens not to attest *kyn* (i.e. if *c*(kyn)=0), SRILM backs off to the 2-gram level to assign probability to it; that is to say, it substitutes *c*(ky)/ *c*(k) × *c*(yn)/ *c*(y) for *c*(kyn)/ *c*(ky).

It is worth noting that unattested 3-grams (and, by extension, any unattested n-gram irrespective of its length) will not all be assigned the same probability. The reason is that this assigning of probability to an unattested n-gram is based on the frequencies of shorter n-grams that are attested. Another thing worth noting is that training-text n-grams irrelevant to the test text *#kyng#* are not part of the formula. Furthermore, our approach is in part a ‘bag of words’ one in the sense that only the longer n-grams include spaces between words, and it matters less **where** in the training text the relevant n-grams occur. This solves the respective problems of variation in word order and differences in the number of words recorded.

### Weights and n-grams to ignore

We introduce weights into the formula to increase the contribution of the longer n-gram models to the perplexity computations while reducing that of the short n-gram ones (the “-mix-lambda” option in code sample below). Simply put, 5-gram matches are made to count relatively more than 4-gram matches and in turn, 4-gram matches more than 3-gram matches, etc. This step prioritizes lexical similarity in perplexity computations at the expense of spelling similarity. The determination of optimal weights was achieved through trial and error. If the tool is applied to a different type of data, the weights may have to be different.

A further step we have taken is to ignore the 50 most frequent spelling forms in the database due to their limited discriminatory power. While one might expect the highly frequent forms on the exclude-list to be function words such as prepositions (OF, IN), conjunctions (AND, BUT), and pronouns (IT, THEY), this expectation is not met exclusively. Some spellings of lexical words such as *god, crist, gospel, men* and *day* also rank in the top-50 (while other spelling forms of these same words do not). Moreover, ME has characteristics that differ from PDE, such as the rarity of both the preposition AT and a *wh-* pronoun like WHO. For this reason, one should be careful about drawing on one’s knowledge of PDE when compiling a list of spelling forms to ignore.

### Smoothing

Furthermore, we invoke Witten-Bell smoothing (“-wbdiscount” in code sample below; Witten & Bell, 1991) both to address the situation where a test text contains an n-gram that is unattested in the training text and to facilitate the transition from a model of the training text itself to a model of the language the training text is a sample of. Smoothing is a standard step in language modelling ( Jurafsky & Martin, 2008, 97–103) Witten-Bell smoothing modifies the attested n-gram frequencies by squaring in the number of unique n-grams that follow the given n-gram. Various other smoothing techniques exist, and they have been experimentally shown to perform equivalently with training texts that are as sparse as the present ones ( Chen & Goodman, 1996; Chen & Goodman, 1999). Moreover, our selection of Witten-Bell smoothing was pragmatically determined: the SRILM toolkit may produce various warnings when other smoothing methods are applied on extremely sparse training data. ^3^

Smoothing methods seek to model the language behind a sample, rather than the sample itself—a valid comparison is with regression modelling, where the goal is to avoid overfitting the model. The aim is instead to capture general properties of the population from which the data set was extracted, rather than specific properties of the data set.

To model a language, the effects of the exact n-grams that constitute the sample need to be reduced. For example, it is reasonable to maintain that 1-grams attested just once in a sample are attested by accident. Different 1-grams would likely be attested just once in another sample of the same language. A 1-gram that happens to be unattested in a training text may, consequently, be assigned the absolute frequency 1 when we back off to the 1-gram level in calculating perplexity in accordance with the formula provided earlier. Smoothing through this process, by adding one to the numerator, solves the problem that backoff to the 1-gram level will not work with unattested 1-grams (since an unattested 1-gram means a 0 appears in a numerator in the formula). The probability corresponding to an absolute frequency of 1 will be infinitesimal with a sizable training text but will not be negligible with the present data because of its extreme sparsity.

Conversely, it makes sense to hold that it is the n-grams that are attested multiple times that best characterise a language. However, with a training text yielding extremely sparse data “multiple times” amount to no more than a handful of instances (bearing in mind that the average incipit is 51 characters long, while the average explicit is 56 characters long, including spaces). Smoothing too little, then, means risking modelling the sample rather than the language, which in turn may mean that two samples of the same language will not be recognized as such. On the other hand, smoothing too much could mean all training texts come to be regarded as samples of one and the same language. The challenge for us, therefore, has been to smooth just enough successfully to model the language rather than the sample; all the while that languages should be kept apart.

Trial and error has taught us that the shortest training texts simply do not yield sufficient information for successfully modelling them, by which we mean for telling them apart. Thus, we have excluded incipits and reverse explicits with less than 35 characters in length.

### The fuzzy search script: A two-step process

Searching can be described as a trade-off between **precision** and **recall**, which are commonly used performance metrics in machine learning (see, for example, VARD2). Precision is the percentage of relevant retrieved matches out of all retrieved matches, whereas recall is the percentage of relevant retrieved matches out of all relevant matches ( Olson & Delen, 2008: 138). The search tool implemented in our resource follows a two-step process, primarily optimised for recall rather than precision, utilizing the methods described above. It retrieves several possible matches for a test text without attempting to select the ‘correct’ one among them. Consequently, the approach prioritises recall over precision, providing scholars with a list of matches to work with. The aim is to have something akin to a Google search, in which the best matches are displayed first, followed by less precise ones.

When a user inputs a test text as their query string, the web application performs two simultaneous tasks. It looks up the query in the database of incipits or reverse explicits (as appropriate) to find exact matches and calls the fuzzy search script with the query string.

The fuzzy script, working in tandem with the web application, is written in Python and employs the SRILM toolkit to find fuzzy matches between the query string and the incipits or explicits in the database. There are two ways to find these matches:

a) Training five models (one per n-gram length, 1-5) on the query string and interpolating them to make them in effect a single model. The model is then tested separately on each separate database record.
b) Training five models (one per n-gram length, 1-5) on each individual database record and then interpolating them to make them in effect into a single model per record. Each such single model is then tested separately on the query string, reversing the roles of training data and test data in comparison with alternative a). ^4^

Manual evaluation of fuzzy matches from queries has revealed that the best matches are obtained by employing alternative b), i.e., by treating the query string as the test text and testing it against each incipit or explicit-based interpolated LM in turn. This alternative also makes the most intuitive sense since incipits and explicits are generally longer than the query string, providing more reliable statistics for building LMs.

However, this approach poses a practical problem, as testing the query string on each interpolated LM separately takes an unreasonably long time (minutes when testing against our current selection of 9676 incipits on a 2.90GHz Intel Xeon Platinum 8268 CPU). Our solution is to apply alternative a) first, since SRILM can test a large number of test texts against a single LM very quickly. As a second step, we then apply alternative b) but only on those database records that show the lowest perplexity value in the first step.

We have built the models drawn upon in alternative b) in advance, whereas SRILM builds the alternative a) models when the fuzzy search script is executed. The database of incipits and reverse explicits that comprises the training texts for alternative b) remains static, whereas this is not the case with the user-input query string that constitutes the training text for alternative a).


  # First create a series of models from the query text
  subprocess.run([SRLIM_DIR +'ngram-count','-order','5','-no-sos','-no-eos','-wbdiscount',
         '-text',query_file,'-lm',"{}_5.lm".format(query_model_file)],universal_newlines=True)
  subprocess.run([SRLIM_DIR +'ngram-count','-order','4','-no-sos','-no-eos','-wbdiscount',
         '-text',query_file,'-lm',"{}_4.lm".format(query_model_file)],universal_newlines=True)
  subprocess.run([SRLIM_DIR +'ngram-count','-order','3','-no-sos','-no-eos','-wbdiscount',
         '-text',query_file,'-lm',"{}_3.lm".format(query_model_file)],universal_newlines=True)
  subprocess.run([SRLIM_DIR +'ngram-count','-order','2','-no-sos','-no-eos','-wbdiscount',
         '-text',query_file,'-lm',"{}_2.lm".format(query_model_file)],universal_newlines=True)
  subprocess.run([SRLIM_DIR +'ngram-count','-order','1','-no-sos','-no-eos','-wbdiscount',
         '-text',query_file,'-lm',"{}_1.lm".format(query_model_file)],universal_newlines=True)

  # Then run all incipits against the query models
  process = subprocess.run([SRLIM_DIR +'ngram','-order','5','-no-sos','-no-eos',
               '-lm','{}_5.lm'.format(query_model_file),'-lambda','0.6',
               '-mix-lm2','{}_4.lm'.format(query_model_file),'-mix-lambda2','0.45',
               '-mix-lm3','{}_3.lm'.format(query_model_file),'-mix-lambda3','0.2',
               '-mix-lm4','{}_2.lm'.format(query_model_file),'-mix-lambda4','0.04',
               '-mix-lm5','{}_1.lm'.format(query_model_file),'-mix-lambda5','0.01',
               '-debug','1','-ppl','/tekstlab/imep/{}s.text'.format(prose_type)],
               stdout=subprocess.PIPE,universal_newlines=True,encoding='UTF-8')

  output_lines = process.stdout.splitlines()
  all_chunks = [ output_lines[i:i +4] for i in range(0,len(output_lines),4) ]
  chunks = all_chunks[:len(all_chunks)-1]
[...]
  incipit_numbers_and_pp1s = [ process_chunk(chunk_index +1,chunk)
                  for (chunk_index,chunk) in enumerate(chunks)
                  if long_enough(incipit_lines[chunk_index]) ]

  # Sort all incipits based on their ppl1 values from the reverse matching
  incipit_numbers_and_pp1s.sort(key=lambda elm: float(elm[1]))

  # Select the best candidates for use in the proper testing procedure
  candidates = incipit_numbers_and_pp1s[:NUM_CANDIDATES]
[...]

  # Now run the query text against each of the NUM_CANDIDATES incipits with the lowest perplexity value
  # in the reversed matching process above,and return the NUM_SELECTIONS ones with the lowest value
  # when we do the 'proper' matching.
  candidates_with_proper_pp1s = []
  for incipit_info in candidates:
[...]
       process = subprocess.run([SRLIM_DIR +'ngram','-order','5','-no-sos','-no-eos',
                    '-lm','{}/{}_5.lm'.format(MODEL_DIR,incipit_number),'-lambda','0.60',
                    '-mix-lm2','{}/{}_4.lm'.format(MODEL_DIR,incipit_number),'-mix-lambda2','0.45',
                    '-mix-lm3','{}/{}_3.lm'.format(MODEL_DIR,incipit_number),'-mix-lambda3','0.2',
                    '-mix-lm4','{}/{}_2.lm'.format(MODEL_DIR,incipit_number),'-mix-lambda4','0.04',
                    '-mix-lm5','{}/{}_1.lm'.format(MODEL_DIR,incipit_number),'-mix-lambda5','0.01',
                    '-ppl',query_file],
                    stdout=subprocess.PIPE,universal_newlines=True,encoding='UTF-8')
       m = re.search(ppl1_pattern,process.stdout.splitlines()[1])
       # Append a tuple containing the incipit number and ppl1 value from the proper matching of the query text against this incipit
       candidates_with_proper_pp1s.append((incipit_number,m.group(1)))
     
  # Sort the candidates based on their proper ppl1 values
  candidates_with_proper_pp1s.sort(key=lambda elm: float(elm[1]))
  selected = list(map(lambda candidate: candidate[0],candidates_with_proper_pp1s))[:NUM_SELECTIONS]


It is important to note that when we refer to having an LM for a query string or an incipit or explicit, we are actually referring to a series of models for different *n* values, ranging from 1-grams to 5-grams. These models are weighted differently when testing a string against them: 5-grams are assigned a weight of 0.5, 4-grams are weighted as 0.4, 3-grams as 0.1, 2-grams as 0.07, and 1-grams as 0.03. As mentioned above, this weighting scheme serves to emphasize larger n-grams while still allowing us to back off to smaller n-grams when the larger ones are unattested in the training data.

The process can be summarised in the following steps:

- Use the ‘ngram-count’ SRILM command to create an LM with the query string as training data.
- Run the ‘ngram’ SRILM command to test a text file containing all database records, each on a separate line, against the search-string LM. The perplexity value for each is recorded. We use the *ppl1* value calculated by SRILM, which does not include end-of-sentence tags.
- Select the 100 records with the lowest *ppl1* value from the previous step and identify the pre-trained LMs for each of them.
- Run the ‘ngram’ command once for each of these LMs, this time using the query as the test string.
- Select the 20 database records with the lowest *ppl1* value from the previous step and return their database IDs and *ppl1* values to the web application.

In addition, the web application, developed with the Django framework and Docker, performs a search directly in the database for the search string. ^5^ The results obtained from the direct database query are combined with the results from the fuzzy search script. The final results are presented as a list by the web application, with exact matches displayed at the top, followed by the fuzzy matches arranged in order of ascending perplexity. This process ensures that the web application prioritises exact matches.

## Discussion

In conclusion, our project introduces a search tool that has been specifically developed to handle the extensive range of variation found in ME. The kinds of variation present in ME incipits, explicits and texts in general are both linguistic and textual. The process of scribal copying leads to non-identical copies of the same work, as evidenced by our Table 1 with incipits from the *Brut* chronicle. Furthermore, the lack of a standardised writing system contributes to this variability. It is perfectly common for a single scribe to employ variable spellings, even spelling multiple occurrences of the same word differently within a few lines. Such variability poses challenges for automatic search tools.

While a simple search combined with regular expressions will yield results that are precise in the sense of containing that specific string, it may not recall every relevant match since some relevant matches will not contain that specific string but rather some variation of it. Repeated searches for specific strings might bring the user closer to recalling every match, but there is no guarantee of reaching the target.

Our approach is the opposite: we deliberately lower precision and make the search tool return ‘too many matches’. These matches will include variants the user will not have predicted. We assume our target audience of manuscript scholars are happy with a shortlist of *potential* matches. We find that users can easily separate matches relevant to them from ones relevant only in the eyes of the search tool. This is advantageous for a tool like the IMEP, enabling scholars to encounter texts they might not have been aware of.

### Weaknesses

One limitation of developing an efficient search tool is the absence of an objective test of precision and recall. While the quality of a machine-translated text can be assessed using a score like BLEU, in which the edit distance between the machine-translated text and a human-translated reference text is used as a quality measure, we only have subjective measures available. We rely on experts in the field to assess the quality of our results, and we let the results of simple string searches inform our assessment of which texts ought to appear among the matches. The current exclude-list and weights attached to each gram-length lead to satisfactory results. However, adjustments to these parameters, as well as the inclusion of 6-grams, could potentially lead to stronger outcomes.

The tool occasionally ranks high an incipit or explicit which is orthographically similar to the search string but lexically different. While such matches rarely rank above every lexically similar match, they may rank higher than some of them. These matches will come across as false positives to human users. This happens more often with one- or two-word search strings, a consequence of the extreme sparsity of the data. A two-word search string will contain fewer than 15 characters while a typical incipit/explicit will be 50–60 characters long. Since strings of these lengths yield little information from which to compute similarity metrics with any confidence, single characters can disproportionately affect model perplexity. Nonetheless, it is not difficult for human users to distinguish between genuine matches and false ones based on similar orthography.

### Future steps

There are many ways in which the resource could be improved. One potential improvement involves further database annotation, which is somewhat limited at the present stage. However, this limitation is intrinsic to the current stage of the project, since the overall aim of IMEP is unifying handlist indexes into “one synoptic tool” ( Sawyer, 2018: 508). Thus, incorporating information akin to that available in DIMEV to facilitate database lookups could be a feasible future direction.

Another possible avenue of future endeavour would be adding lemmatising. Researchers have generally shied away from developing lemmatisation software for ME, and no lemmatiser is currently available on the market.

Access to medieval manuscripts is dependent on the search tools available to us. We do not yet have an exhaustive record of what ME works exist and how they relate to one another. Nonetheless, the use of perplexity of n-gram language models as a similarity metric has significant potential in studies of ME texts. Our tool is downloadable and modifiable for use with similar datasets.

## Data Availability

The development of the search tool is a part of the MSCA IF action *Index of Middle English Prose: Digital Cotton Catalogue Project* (IMEP DCCP), of which the principal author has been the beneficiary. Following the data management plan of the MSCA IF action IMEP DCCP, longer manuscript descriptions written as a part of the project will be published as a printed manuscript catalogue by the publisher Boydell & Brewer. For contractual reasons, the printed volume is subject to copyright by the publisher. This is standard practice for the IMEP handlists. Short incipits and explicits of the catalogue are made available OA through the IMEP website hosted at the CUL. The CUL website is open to the public, granting unrestricted access without any associated fees. The source code for the website is maintained by the CUL.

## Footnotes

^1^ The encoding uses the following special characters for graphs used in ME: &thorn; - þ, &eth; - ð, &wynn; - ƿ, &yogh; - ȝ. &aelig; - æ.

^2^ Perplexity was defined as the “weighted average branching factor” above, which is another way of saying the same thing.

^3^ As is acknowledged on its FAQ pages ( http://www.speech.sri.com/projects/srilm/manpages/

^4^ Note: the actual interpolation takes place during the testing of the models rather than during the construction of the models.

^5^ https://www.djangoproject.com/

https://www.docker.com

## Software availability

The search tool makes use of the language modelling software SRILM (SRI Language Modeling toolkit). According to SRILM’s Terms of Use: Government agencies, and schools, universities, and non-profit organizations can download SRILM free of charge under SRI's "Research Community License", for use in projects that do not receive external funding other than government research grants and contracts.

The source code of SRILM is freely available on GitHub. https://github.com/BitSpeech/SRILM

https://open-research-europe.ec.europa.eu/for-authors/data-guidelines/

Source code available from: GitHub: noklesta/imep_fuzzy_search

Archived source code at time of publication: 10.5281/zenodo.8396406 Nøklestad (2023)

License: MIT
