# Supplementary material for: A search tool based on language modelling developed for The Index of Middle English Prose
Source: Open Res Eur. 2024 Mar 11;3:197. Originally published 2023 Nov 14. [Version 2] doi: 10.12688/openreseurope.16590.2 (PMC10808851; doi:10.12688/openreseurope.16590.2)
Supplement: Peer review history

# Reviewer report: Reviewer response for version 2

**DOI:** 10.21956/openreseurope.18892.r38851

**Published:** 2024-04-22

**Authors:** Cristina Marras

**Affiliations:** 1 Italian National Research Council, Rome, Italy

No further comments.

Are the conclusions about the tool and its performance adequately supported by the findings presented in the article?

Yes

Is the rationale for developing the new software tool clearly explained?

Yes

Is the description of the software tool technically sound?

Yes

Are sufficient details of the code, methods and analysis (if applicable) provided to allow replication of the software development and its use by others?

Yes

Is sufficient information provided to allow interpretation of the expected output datasets and any results generated using the tool?

Yes

Reviewer Expertise:

Digital Humanities; Lexicography; History of Philosophy; Philosophy of Language.

I confirm that I have read this submission and believe that I have an appropriate level of expertise to confirm that it is of an acceptable scientific standard.

# Reviewer report: Reviewer response for version 1

**DOI:** 10.21956/openreseurope.17907.r36581

**Published:** 2024-01-24

**Authors:** Cristina Marras

**Affiliations:** 1 Italian National Research Council, Rome, Italy

The article clearly addresses some of the problem of the search tools used in non-standardised early vernaculars. In particular focuses on the high degree of variation in orthography, syntax, and lexicon between titles, incipits, and explicits detected in manuscript copies.

 Authors present a software available as a part of the *Index of Middle English Prose* (IMEP) specifically design to cope with these variations.

 In the paper the developed tool is clearly described, it uses  a database character-based n-gram language models (LMs) built with the *Stanford Research Institute Language Modelling * (SRILM) toolkit, and a fuzzy search script (IMEP: FSS) written in Python. The search process, steps, approaches, tools and web applications are discussed and and are clearly presented in their functionality.

 The part dedicated to the critical and weak aspects of the instrument and the proposals for development and improvement are appreciated. As well as appreciating the availability of software in open access and its adaptability to other database.

Are the conclusions about the tool and its performance adequately supported by the findings presented in the article?

Yes

Is the rationale for developing the new software tool clearly explained?

Yes

Is the description of the software tool technically sound?

Yes

Are sufficient details of the code, methods and analysis (if applicable) provided to allow replication of the software development and its use by others?

Yes

Is sufficient information provided to allow interpretation of the expected output datasets and any results generated using the tool?

Yes

Reviewer Expertise:

Digital Humanities; Lexicography; History of Philosophy; Philosophy of Language.

I confirm that I have read this submission and believe that I have an appropriate level of expertise to confirm that it is of an acceptable scientific standard.

# Reviewer report: Reviewer response for version 1

**DOI:** 10.21956/openreseurope.17907.r36261

**Published:** 2023-12-15

**Authors:** Ondrej Tichy

**Affiliations:** 1 Department of the English Language and ELT Methodology, Charles University, Prague, Czech Republic

The article presents an innovative digital search tool designed to handle linguistic and textual variation in non-standardized early vernaculars, such as Middle English. The tool addresses challenges in searching texts due to variations in orthography, syntax, and lexicon found in manuscript copies of the same work, an issue not adequately addressed by traditional search methods like exact string matching, regular expressions, dictionary or edit distance based standardization. The core of the tool is based on the Stanford Research Institute Language Modelling (SRILM) toolkit that is leveraged by a fuzzy search script (IMEP: FSS) written in Python. The article showcases an implementation of the tool on the data of the Index of Middle English Prose (IMEP) allowing users to find copies of the same texts by they highly varied incipits and explicits. Based on the results presented in the paper, the authors were very successful in this implementation and it can be expected their tool will find more use with other datasets as well.

 There are only a couple of minor suggestions to this version of the paper:

 1. When discussing the limitations of commonly used computational approaches (p. 7), it might be worthwhile to also consider their combinations.

 2. The definitions of incipits and explicits should come earlier than p. 7. This may be important to non-medievalist audience.

 3. If one of the upshots of the process summary presented on p. 11 (esp. its third point) is that with its current setting the tool will provide users with max. 100 results, this might be stated explicitly.

 4. At the top of p. 12, it is not quite clear whether the search is for a regexp or exact match or what role the regexp plays in it.

Are the conclusions about the tool and its performance adequately supported by the findings presented in the article?

Yes

Is the rationale for developing the new software tool clearly explained?

Yes

Is the description of the software tool technically sound?

Yes

Are sufficient details of the code, methods and analysis (if applicable) provided to allow replication of the software development and its use by others?

Yes

Is sufficient information provided to allow interpretation of the expected output datasets and any results generated using the tool?

Yes

Reviewer Expertise:

historical linguistics, history of English, spelling variation, digital humanities, historical lexicography

I confirm that I have read this submission and believe that I have an appropriate level of expertise to confirm that it is of an acceptable scientific standard.

# Response

**Published:** 2024-03-07

**Authors:** Alpo HONKAPOHJA

We thank the reviewer for the detailed review and insightful comments. We have addressed all comments in the revised version except for comment 1, which suggests improving precision by combining multiple string similarity metrics. We have opted not to do so because addressing this point would considerably lengthen the discussion of alternative approaches, which is already fairly long. Moreover, while combining multiple strings could potentially improve precision, doing so would also likely slow down searches considerably. Users may not tolerate search times lasting several seconds in a web-based search tool. The two-step matching process used by the fuzzy search script was specifically developed to address the issue of overly long search times. In the future, with increased computing power, it is likely that computing multiple string similarity metrics will become viable.
